# Association of Polymorphisms in *miR146a*, an Inflammation-Associated MicroRNA, with the Risk of Idiopathic Recurrent Spontaneous Miscarriage: A Case-Control Study

**DOI:** 10.1155/2022/1495082

**Published:** 2022-04-30

**Authors:** Saeedeh Salimi, Saman Sargazi, Behrouz Mollashahi, Milad Heidari Nia, Shekoufeh Mirinejad, Mahdi Majidpour, Marzieh Ghasemi, Sara Sargazi

**Affiliations:** ^1^Department of Clinical Biochemistry, School of Medicine, Zahedan University of Medical Sciences, Zahedan, Iran; ^2^Cellular and Molecular Research Center, Research Institute of Cellular and Molecular Sciences in Infectious Diseases, Zahedan University of Medical Sciences, Zahedan, Iran; ^3^Genetics of Non-Communicable Disease Research Center, Zahedan University of Medical Sciences, Zahedan, Iran; ^4^Pregnancy Health Research Center, Zahedan University of Medical Sciences, Zahedan, Iran; ^5^Moloud Infertility Center, Ali ibn Abitaleb Hospital, Zahedan University of Medical Sciences, Zahedan, Iran; ^6^Faculty of Medicine, Zahedan University of Medical Sciences, Zahedan, Iran

## Abstract

It has been established that microRNAs (miRNAs) are involved in the regulation of immune responses and serve as biomarkers of inflammatory diseases as well as recurrent spontaneous miscarriage (RSM). Herein, we aimed to study the relationship between three functional *miR146a* gene polymorphisms with idiopathic RSM (IRSM) susceptibility. We recruited 161 patients with IRSM and 177 healthy women with at least one live birth and without a history of abortion. Genotyping was performed using RFLP-PCR and ARMS-PCR methods. We found that the rs6864584 T/C decreased the risk of IRSM under dominant TT+TC vs. CC (OR = 0.029) and allelic C vs. T (OR = 0.028) contrast models. Regarding rs2961920 A/C and rs57095329 A/G polymorphisms, the enhanced risk of IRSM was observed under different genetic contrasted models, including the codominant CC vs. AA (OR = 2.81 for rs2961920) and codominant GG vs. AA (OR = 2.36 for rs57095329). After applying a Bonferroni correction, haplotype analysis revealed a 51% decreased risk of IRSM regarding the ACA genotype combination. This is the first study reporting that *miR146a* rs57095329 A/G, rs2961920A/C, and rs6864584 T/C polymorphisms are associated with the risk of IRSM in a southern Iranian population. Performing replicated case-control studies on other ethnicities is warranted to outline the precise effects of the studied variants on the risk of gestational trophoblastic disorders.

## 1. Introduction

Recurrent spontaneous miscarriage (RSM) is defined as the occurrence of 3 consecutive losses < 20 weeks gestation [1]. This condition has a fascinating yet poorly understood etiology. It affects about 5% of couples; due to its complex and often undefined etiology, RSM has been a tremendous challenge in reproductive medicine [1, 2]. Exposure to environmental factors, genetics and hormonal abnormalities, immune reactions, embryo-fetal infections, uterine pathologies, antiphospholipid syndrome, sperm oxidative stress, drug abuse, and psychological trauma have been implicated as etiologic factors of RSM [3–5]. However, 40-60% of the RSM cases have no specific pathology and thus are classified as unexplained or idiopathic RSM (IRSM) [6]. Lately, several single nucleotide polymorphisms (SNPs) have been associated with IRSM occurrence [7–9]. Of those, SNPs that resided in genes encoding microRNAs (miRNAs or miRs) have been brought to the attention [10]. Essentially, a significant proportion of IRSM might be due to SNPs in coding- or noncoding genome [11].

Thus far, growing evidence has demonstrated that deregulated miRNA expression is associated with female reproductive diseases, ranging from repeated implantation failure to IRSM [12, 13]. MicroRNAs (miRNAs), as single-stranded RNA molecules of approximately 20-22 nucleotides, act as posttranscriptional regulators of gene expression by translational repression or messenger RNA degradation [14]. Today, it has been established that a single miRNA regulates several targets and therefore acts as a master control of gene expression. Even though miRNAs comprised less than 3% of the human genome, bioinformatics tools have shown that nearly 30% of human genes are targeted by these noncoding RNAs [14], indicating the important function of miRNAs in both pathological and physiological processes. Many miRNAs were already associated with RSM [15]. Among those, miR165a-3p, miR408-5p, miR1143-3p, miR4235, miR5386, miR5813, miR5525, miR4407, miR5567, etc., were differentially expressed between normal and abnormal embryos [16]. Besides, while miR-517c, miR-519a-1, miR-522, miR-520h, miR-184, miR-187, and miR-125b-2 were upregulated in RSM, miR-520f, miR-3175, and miR-4672 were downregulated in RSM [17].

As an inflammation-associated miRNA, miR146a is a candidate miRNA found dysregulated in different diseases, including tumors, coronary artery disease, periodontal disease, epilepsy, and, as previously shown, preeclampsia [18–22]. Moreover, miR146a mediates inflammatory pathways in preterm birth patients [23]. It has been shown that miR146a mediates inflammatory changes and tissue fibrosis [24]. Su et al. reported that administration of scavenger receptor/Toll-like receptor 9 agonist (C-miR146a) suppressed the expression of nuclear factor-kappa light chain enhancer of activated B cell (NF-*κ*B) target genes [25]. Upregulation of *miR146a* has been associated with inhibiting inflammatory responses in lipopolysaccharide- (LPS-) induced acute lung injury [26].

Mutations alter the total expression and prematurity processing of *miR-146a* and affect susceptibility to multiple diseases [27]. Functional *miR146a* variants, rs57095329 A>G [minor allele frequency (MAF): 0.021-0.5], rs2961920A/C (MAF: 0.168-0.453), and rs6864584 T/C (MAF: 0.001-0.392) reside in the promoter of the *miR146a* precursor gene. It has already been established that rs2910164 G>C and rs57095329 A>G polymorphisms can potentially alter *miR146a* function and/or expression [28].

For all we know, no study has integrated these findings into the relationship between these SNPs and IRSM. Hence, in this preliminary study, we aimed to examine the relationship between three *miR-146a* SNPs (rs2961920A/C, rs57095329 A/G, and rs6864584 T/C) with IRSM incidence in a south-east Iranian population.

## 2. Subjects and Methods

### 2.1. Participants

The study group consisted of 161 women with idiopathic IRSM, who presented with a history of at least two consecutive pregnancy losses at less than 20 weeks of gestation and 177 healthy women as controls. The enrollment procedure has been described in our previous work [29]. The local Ethics Committee of Zahedan University of Medical Sciences approved the study (IR.ZAUMS.REC.1399.093). Informed consent was taken from all participants.

### 2.2. DNA Extraction and Genotyping

The salting-out method was employed to extract DNA from the whole venous blood of participants. A NanoDrop spectrophotometer (Thermo Fisher Scientific, USA) was used to verify the quantity and purity of the extracted DNAs. Electrophoresis on agarose gel was carried out to evaluate the quality of extracted DNA.

**Figure 1:**
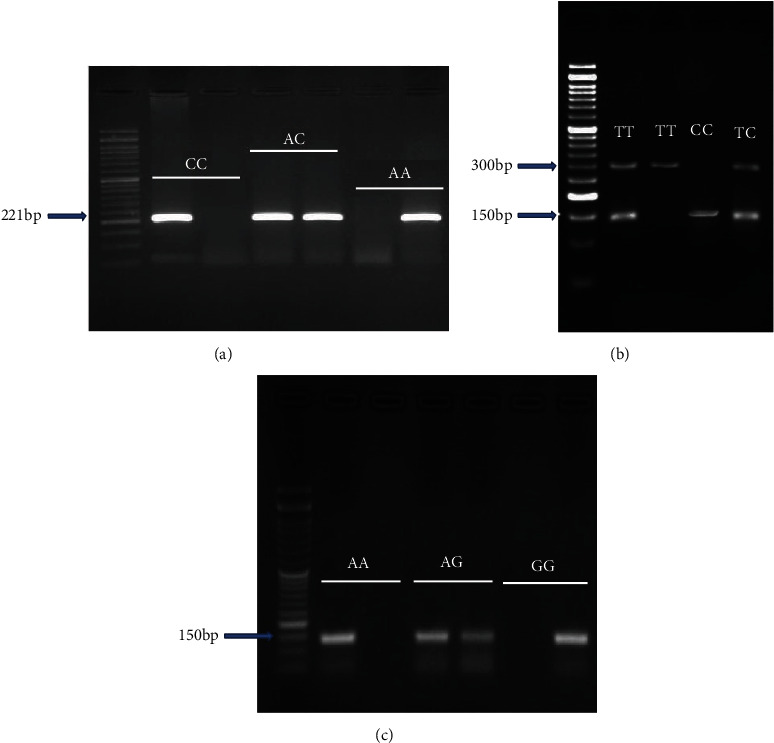
Gel photograph of PCR amplification products of the *miR146a* (a) rs2961920A/C, (b) rs6864584 C/T, and (c) rs57095329 A/G polymorphisms.

Genotyping of rs6864584 polymorphisms was performed via both polymerase chain reaction-restriction fragment length polymorphism (PCR-RFLP). The used primer sequences were already reported in Sargazi et al.'s study [30]. For detecting all three SNPs, PCR was performed in a total volume of 15 *μ*L, containing 1 *μ*L (100 ng/*μ*L) genomic DNA, 1 *μ*L of each primer (6 pmol), 10 *μ*L Taq DNA Polymerase 2X Master Mix Red (Ampliqon, Denmark), and 2 *μ*L of distilled water. The PCR conditions were as follows: denaturation at 95°C for 5 min, 35 cycles at 95°C for 35 s, annealing temperature (according to Table 1 for each SNP) for 30 s and 72°C for 45 s, and a final extension at 72°C for 5 min. PCR products were electrophoresed on 1.5% agarose gel and stained in 0.25 *μ*g/mL ethidium bromide, and bands were visualized (Figure 1). The C allele of rs6864584 was digested by the *ApaI* and produced a 152- and 148 bp band, and the undigested T allele has a 300 bp fragment. Regarding rs57095329 and rs2961920, we used allele-specific amplification-refractory mutation system-polymerase chain reaction (ARMS-PCR) [31] in two well-defined conditions. For this purpose, the forward primer for one allele and the common primer were placed in one well. At the same time, the other forward primer specific for the other allele and the common primer were added to another well, and PCR reaction was performed following the condition above. In the presence of either the homozygous genotypes, the amplified band will be visualized in separate wells. In contrast, the bands can be visualized in both wells in the presence of a heterozygous genotype. The amplicon sizes for genotyping rs57095329 and rs2961920 were 153 and 221 bp, for both A and G or A and C alleles, respectively (Table 1). The bands were then displayed via agarose gel electrophoresis (Figure 1). About 30% of the samples were randomly regenotyped for quality control, and the reproducibility of the results was 100%.

### 2.3. Statistical Analysis

The SPSS 23.0 software (IBM Corporation, Armonk, NY) was used for data analysis. Deviations from Hardy Weinberg equilibrium (HWE) were examined using a chi-square test. Fisher's exact test was used to compare differences between data sets. The association between the alleles and genotypes of *miR146a* polymorphisms and IRSM risk was examined by odds ratios (ORs) and 95% confidence intervals (CIs), using unconditional logistic regression analysis. The SHEsis software platform was used to determine the amount of linkage disequilibrium (LD) between the studied SNPs [32]. Statistical significance considered as a *p* value < 0.05.

## 3. Results

The subject's ages differed from 15 to 55 years, with the mean age of 31.6 ± 6.8 (in controls) and 32.5 ± 6.6 (in IRSM patients). The mean (SD) BMI of controls was 26.2 ± 4.4 versus 26.3 ± 4.7 in cases. We found no marked difference between cases and controls regarding age (*p* = 0.23) and body mass index (BMI, *p* = 0.77). For all the studied SNPs, we did not observe any departure from HWE in healthy women (*p* value for HWE > 0.05 as shown in Table 2), indicating the proper selection of the controls.

Genotypic and allelic distribution of *miR146a* SNPs is presented in Table 2. We found that the AC genotype of rs2961920, the TC genotype of rs6864584, and the AG genotype of rs57095329 were more frequent in controls and selected as the reference genotypes. Both rs57095329 A/G and rs2961920 A/C enhanced the risk of IRSM in our population, whereas alleles and genotypes of rs6864584 C/T showed a protective role against the disease. The C allele of rs2961920 A/C increased the risk of IRSM under codominant CC vs. AA (OR = 2.81, CI (1.34-5.88), and *p* = 0.005), recessive CC vs. AC+AA (OR = 2.77, CI (1.39-5.54), and *p* = 0.003), and allelic C vs. A (OR = 1.41, CI (1.03-1.93), and *p* = 0.032) genetic patterns. In terms of rs57095329 A/G, an increased risk of developing IRSM was found under the codominant GG vs. AA (OR = 2.36), codominant AG vs. AA (OR = 1.87), and dominant AA+AG vs. GG (1.94) modes of inheritance. The G allele of this SNP enhanced the risk of IRSM by 1.52 folds (OR (95%CI) = 1.52 (1.11–2.08), *p* value = 0.011). Regarding rs6864584 C/T, the frequency of the C allele in the control group was higher than cases, suggesting a protective role against IRSM (OR (95% CI) =0.68 (0.48–0.95), *p* value = 0.028). A diminished risk of IRSM was also noticed under the dominant TT+TC vs. CC (OR = 0.61) genetic pattern of this variation.

Table 3 shows the interaction analysis of *miR146a* polymorphisms on IRSM risk. None of the genotype combinations of rs2961920, rs6864584, and rs57095329 polymorphisms significantly affected IRSM risk, after applying a Bonferroni correction for multiple testing (*p* > 0.017).

Table 4 demonstrates the haplotype analysis of *miR146a* SNPs in IRSM patients and healthy women. Our results indicated that the ACA haplotype significantly decreased the risk of IRSM compared with rs2961920A, rs6864584T, and rs57095329T haplotype (OR (95%CI) = 0.49 (0.28–0.85), *p* value = 0.01).

**Figure 2:**
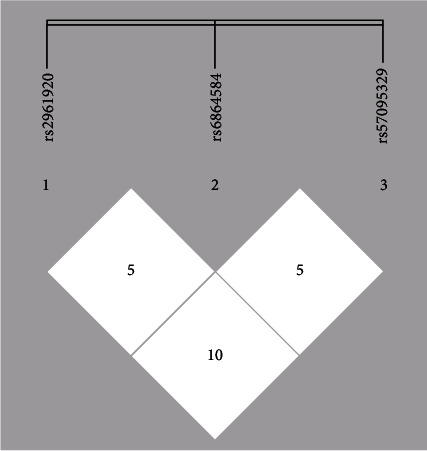
LD analysis of rs2961920A/C, rs6864584 C/T, and rs57095329 A/G polymorphisms. No strong linkage disequilibrium was observed between the studied SNPs.

Linkage disequilibrium (LD) analysis indicated that the cohesion coefficient between rs2961920A/C and rs57095329 A/G was higher than the others. Low D' percentages were noticed between rs6864584 C/T and rs57095329 A/G and also between rs6864584 C/T and rs2961920 A/C polymorphisms (about 5%). Hence, our results suggest no strong linkage between the studied variants in our population (Figure 2).

**Figure 3:**
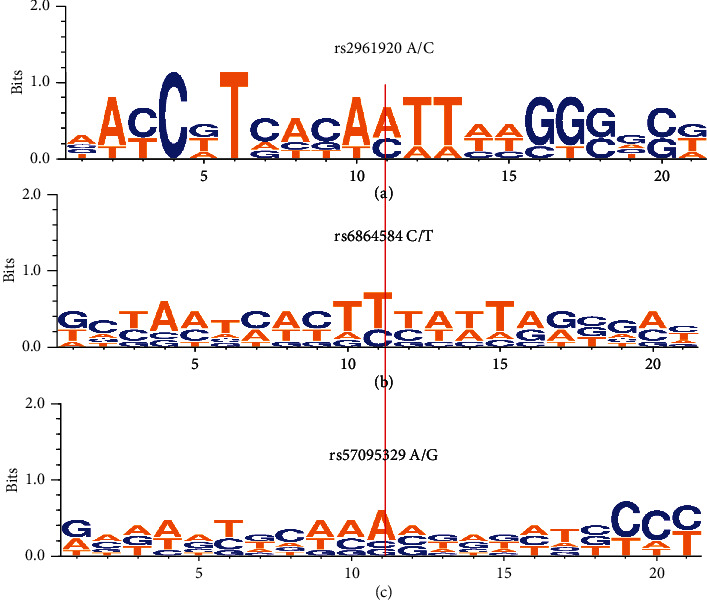
Illustration of sequence conservation by WebLogo around (a) rs2961920A/C, (b) rs6864584 C/T, and (c) rs57095329 A/G SNP locus; human DNA sequences around these loci are presented at the top. The high nucleotides' symbols show more conservation, while the small and more diverse nucleotides indicate less conservation.

We used the WebLogo server to determine the conservation of DNA sequences containing the studied polymorphism sites. As illustrated in Figure 3, *miR146a* rs57095329 A/G and rs2961920 A/C polymorphisms are not located in the conserved regions across multiple mammalian species.

## 4. Discussion

miR146a has a pivotal role in modulating inflammatory responses, as it negatively modulates the inflammatory cytokines, including interleukin 6 (IL-6) and interleukin 6 (IL-8) [33], T-helper 17 (Th17) [34], T-helper 1 (Th1) [35], and NF-*κ*B pathway [36]. Genetic variations in the *miR146a* gene have been correlated with the risk of different diseases. We had already shown that different haplotypes and genotypes of *miR146a* polymorphisms could influence the odds of developing chronic kidney disease, another inflammatory condition [30]. However, no study has reported the link between these functional *miR146a* polymorphisms and IRSM in the Iranian population. The current study's findings demonstrated that alleles and genotypes of *miR146a* SNPs were associated with the risk of IRSM. Besides, we found that a haplotype of *miR146a* polymorphisms decreased the disease risk by 51%. Our results suggest that *miR146a* might be a biomarker for IRSM susceptibility.

A plethora of studies has established the link between *miR146a* variations and susceptibility to sepsis [37], lupus erythematosus [28], type 1 diabetes [38], type 2 diabetes [39], coronary heart disease [40], ischemic stroke [41], preeclampsia [21, 42], and different types of cancer including hepatocellular carcinoma [43], prostate cancer [44], breast cancer [45], and cervical cancer [46]. Still, a minority of studies have failed to report a correlation between SNPs in this gene and susceptibility to ischemic stroke [40] and colorectal cancer [47].

As the first report on the Iranian population, Alipour and coworkers demonstrated an association between an SNP located in *miR146a* and IRSM risk in 2019. In this connection, they screened the rs2910164 C/G polymorphism on 120 Iranian women diagnosed with IRSM and 90 controls. They discovered significant associations between this SNP and RSM incidence in CC vs. GG (OR = 0.481, *p* = 0.015) and CG vs. GG (OR = 2.125, *p* = 0.014) genetic models. Besides, individuals who had either heterozygous or homozygous specified polymorphism (carriers of CG+GG genotype) had a 2.077-fold increased RSM risk [48]. The findings of this report have established the role of miR146a, as a circulating RNA, and SNPs located in *miR146a* in the pathophysiology of IRSM. Two years later, Babakhanzadeh et al. failed to report an association between CC vs. CG+GG (*p* = 0.854) and CC+CG vs. GG (*p* = 0.282) genotypes of miR146a rs2910164 polymorphism and risk of IRSM in women from Yazd Province, located in the center of Iran [49]. Other SNPs in the *miR146a* gene, rs6864584 T/C, rs2961920 A/C, and rs57095329 A/G, were less studied. Still, the findings of some studies support the contribution of these variants to susceptibility to different diseases. Zha et al. found a significant correlation between allele and genotypes of *miR146a* rs57095329 A/G polymorphism and the risk of developing coronary artery lesions in Kawasaki patients. Meanwhile, the *miR146a* rs6864584 T/C was not correlated with the risk of developing Kawasaki disease [50]. In another case-control study, Devanandan et al. proposed that the A_rs57095329_G_rs2910164_ enhances the risk of developing acute lymphoblastic leukemia by fold, whereas the G_rs57095329_G_rs2910164_ represented a protective haplotype for the disease [51].

To this date, no study has reported the possible correlation between these two *miR146a* SNPs with the risk of gestational trophoblastic diseases. There are very limited reports on the correlation between *miR146a* rs2961920 A/C and the risk of a multifactorial disease. In this context, Guo and associates genotyped 502 healthy controls and 529 patients diagnosed with glioma using the Agena MassARRAy Gold technique. Their findings revealed that rs2961920, as a promoteric variant for miR146a and an intronic variant for MIR3142HG, enhanced the glioma susceptibility in Chinese subjects (OR = 1.53, *p* = 0.019) [52]. In another study, Oetting et al. showed that the rs2961920, a proxy for rs2910164, can independently predict acute rejection in kidney transplant recipients [53]. In agreement with these reports, we found the enhanced risk of IRSM in Iranian subjects carrying the CC genotype of *miR146a* rs2961920 A/C under codominant CC vs. AA, recessive CC vs. AC+AA, and allelic C vs. A mode of inheritance.

As a circulating miRNA, miR146a controls megakaryocyte differentiation, adaptive immunity, and cancer [54]. On the other hand, completion of pregnancy highly depends on maternal immune tolerance to the fetal-placental unit, where imbalances between some signaling molecules (specifically Th1- and Th2-type cytokines) increase the incidence of miscarriage [55].

The decidual tissue contains different cytokine-producing cells [56]. Zhao et al. showed that *miR146a-5p* is downregulated in the deciduae of RSM patients compared with healthy controls [55]. Moreover, it has been established that miR146a improves the decidual cytokine microenvironment through modulating the Toll-like receptor (TLR) pathway and other possible mechanisms of RSM pathogenesis [57]. Furthermore, Chen et al. proposed that miR146a downregulates *TLR4*, tumor necrosis factor receptor-associated factor-6 (*TRAF6*), and IL-1 receptor-associated kinase 1 (*IRAK1*) and phosphorylates NF-*κ*B. Consequently, suppressing TLR4/TRAF6/JNK and TLR4/NF-*κ*B pathways inhibits proinflammatory cytokine production [58]. This is important since the upregulation of NF-*κ*B and/or TLR4 at the protein or gene level is involved in RSM pathogenesis [59, 60]. Gysler and colleagues reported that transfection of trophoblastic cells with miR146a-5p mimic caused increased IL8 secretion. However, in contrast, patients with adverse pregnancy outcomes express higher levels of *miR146a-3p* than healthy pregnant women experiencing no pregnancy complications [61]. Together, TLR4- and NF-*κ*B-dependent miR-146a expression might contribute to the protective role of *miR-146a* polymorphisms against IRSM.

RSM is a very heterogeneous disease, and various etiologies are implicated in RSM, including factors known to be causative and those implicated as possible causative agents [i.e., genetic causes (aneuploidy, somatic mutations, and SNPs) and immunologic causes]. Autoimmune and alloimmune causes, anatomic causes, uterine Müllerian anomalies, infectious causes, environmental causes, smoking, endocrine factors, diabetes mellitus, and hematologic disorders are the common etiologic factors for recurrent abortion [62–66]. Dysregulation of many genes, including *miR146a,* is important in preserving pregnancy; therefore, SNPs within these genes might predict RSM susceptibility. Although the etiology of RSM is still unclear, discovering candidate SNPs can help understand the pathophysiology of this complex disease. Still, other mechanisms are involved in the pathogenesis of IRSM that remains to be fully elucidated. Such DNA variations might serve as a valuable prognostic factor to predict the risk of developing such gestational diseases and can be exploited in “Personalized Medicine” applications [67, 68].

Despite all the efforts made, there were some limitations to the current study. The main limitation was using a relatively small sample size. Although we could not perform sequencing to validate our results, random regenotyping of samples showed a very high genotyping accuracy. Moreover, an assessment of different hormones in the serum samples of IRSM patients and healthy controls is needed to correlate miR-146a with serum hormones and give a better idea of the clinical significance of this circulating miRNA. Finally, mRNA levels of *miR146a* were not evaluated to determine the link between genotypes of *miR146a* polymorphisms and *miR146a* expression, which requires further study.

## 5. Conclusion

This study first reported that *miR146a* rs57095329 A/G, rs2961920 A/C, and rs6864584 T/C polymorphisms are correlated with the risk of IRSM in a southern Iranian population. These results provide an opportunity to approach the problems of prognosis and diagnosis of RSM. Still, performing replicated case-control studies on other ethnicities is warranted to outline the precise effects of the studied variants on the risk of gestational disorders.

## Figures and Tables

**Table 1:** The primers used for genotyping of *miR146a* gene polymorphisms.

SNP	Primers	Sequence (5′ to 3′)	Annealing temperature	Length of PCR product (bp)	Restriction enzyme
rs2961920 C/A	F (C-allele)	GTGTATAAGGAAGCCTCACGC	58°C	C:221	*-*
	F (A-allele)	GTGTATAAGGAAGCCTCACGA		A:221	
	R (common)	TGAACACACGGACATCTGAC			

rs6864584 C/T	F	CGGAGAGTACAGACAGGAAGC	59.5°C	T: 300	*ApaI*
	R	TAGATCCCTCCTCGGCACAGC		C: 148 + 152	

rs57095329 A/G	F (A-allele)	CGGGACTGCGGAGAGTACGGA	61°C	A:153	*-*
	F (G-allele)	CGGGACTGCGGAGAGTACGGG		G:153	
	R (common)	TTGGAGCACGTGTCAGGAGCAG			

F: forward; R: reverse; PCR: polymerase chain reaction; SNP: single-nucleotide polymorphism.

**Table 2:** Genotypic and allelic distribution of *miR146a* polymorphisms in IRSM patients and controls.

SNP	Type	IRSM (%)	Control (%)	Genetic model	OR (95% CI)	*p* value
rs2961920 A/C	AA	58 (36.0)	73 (41.2)	Codominant AC vs. AA	1.02 (0.64-1.62)	0.921
	AC	74 (46.0)	91 (51.5)	Codominant CC vs. AA	2.81 (1.34-5.88)	0.005
	CC	29 (18.0)	13 (7.3)	Dominant CC+AC vs. AA	1.25 (0.80-1.93)	0.325
	HWE	0.865	0.073	Recessive CC vs. AC+AA	2.77 (1.39-5.54)	0.003
	A	190 (59.0)	237 (66.9)	Overdominant AC vs. AA+CC	0.80 (0.52-1.23)	0.317
				Allelic	1 [reference]	
	C	132 (41.0)	117 (33.1)	Allelic	1.41 (1.03-1.93)	0.032

rs6864584 C/T	TT	91 (56.5)	78 (44.1)	Codominant CT vs. TT	0.63 (0.40-0.99)	0.056
	TC	61 (37.9)	83 (46.9)	Codominant CC vs. TT	0.48 (0.20-1.15)	0.146
	CC	9 (5.6)	16 (9.0)	Dominant TT+TC vs. CC	0.61 (0.39-0.93)	0.029
	HWE	0.768	0.358	Recessive CC vs. TC+TT	0.60 (0.26-1.39)	0.316
				Overdominant TC vs. TT+CC	0.69 (0.44-1.07)	0.118
	T	243 (75.5)	239 (67.5)	Allelic	1 [reference]	
	C	79 (24.5)	115 (32.5)	Allelic	0.68 (0.48-0.95)	0.028

rs57095329 A/G	AA	45 (27.9)	76 (42.9)	Codominant AG vs. AA	1.87 (1.17-2.99)	0.013
	AG	95 (59.0)	86 (48.6)	Codominant GG vs. AA	2.36 (1.11-5.05)	0.039
	GG	21 (13.1)	15 (8.5)	Dominant AA+AG vs. GG	1.94 (1.23-3.06)	0.006
	HWE	0.008	0.171	Recessive GG vs. AG+AA	1.62 (0.80-3.26)	0.237
				Overdominant AG vs. AA+GG	1.52 (0.99-2.34)	0.070
	A	185 (57.5)	238 (67.2)	Allelic	1 [reference]	
	G	137 (42.5)	116 (32.8)	Allelic	1.52 (1.11-2.08)	0.011

SNP: single-nucleotide polymorphism; CI: confidence interval; OR: odds ratio; IRSM: idiopathic recurrent spontaneous miscarriage; HWE: Hardy-Weinberg equilibrium. *p* < 0.05 was considered statistically significant.

**Table 3:** Interaction analysis of *miR146a* polymorphisms on IRSM risk.

rs2961920 A/C	rs6864584 C/T	rs57095329 A/G	IRSM (%)	Control (%)	OR (95% CI)	*p* value^∗^
AC	TC	AG	20 (12.4)	29 (16.4)	1 [reference]	
AA	TC	AA	7 (4.3)	20 (11.3)	0.51 (0.18-1.43)	0.194
AA	TC	AG	12 (7.5)	13 (7.3)	1.34 (0.51-3.53)	0.555
AA	TT	AA	12 (7.50)	17 (7.9)	1.02 (0.40-2.60)	0.961
AA	TT	AG	20 (12.4)	13 (7.3)	2.23 (0.91-5.49)	0.079
AA	TT	GG	2 (1.2)	5 (2.8)	0.58 (0.10-3.29)	0.535
AC	CC	AG	3 (1.9)	4 (2.3)	1.09 (0.22-5.39)	0.918
AC	TC	AA	4 (2.5)	12 (6.80)	0.48 (0.14-1.72)	0.255
AC	TT	AA	11 (6.8)	18 (10.2)	0.89 (0.35-2.27)	0.801
AC	TC	AG	21 (13.0)	18 (10.2)	1.69 (0.72-3.95)	0.224
AC	TT	GG	9 (5.6)	3 (1.7)	4.35 (1.05-18.09)	0.034
CC	TC	AG	10 (6.2)	4 (2.3)	3.63 (1.00-13.19)	0.043
CC	TT	AA	6 (3.7)	4 (2.3)	2.17 (0.54-8.71)	0.265
CC	TT	AG	8 (5.0)	2 (1.1)	5.80 (1.11-30.23)	0.024

OR: odds ratio; CI: confidence interval; IRSM: idiopathic recurrent spontaneous miscarriage. ^∗^*p* < 0.017 was considered statistically significant after Bonferroni correction.

**Table 4:** Haplotype analysis of *miR146a* polymorphisms in women with IRSM and healthy women.

rs2961920 A/C	rs6864584 C/T	rs57095329 A/G	IRSM	Control	OR (95% CI)	*p* value^∗^
A	T	T	90 (27.9)	110 (31.2)	1 [reference]	
A	C	A	24 (7.5)	60 (17.1)	0.49 (0.28-0.85)	0.010
A	C	G	21 (6.5)	19 (5.3)	1.35 (0.68-2.67)	0.385
A	T	G	55 (17.1)	47 (13.4)	1.43 (0.89-2.31)	0.142
C	C	A	13 (3.9)	11 (3.0)	1.44 (0.62-3.38)	0.395
C	C	G	21 (6.6)	25 (7.1)	1.03 (0.54-1.95)	0.936
C	T	A	58 (18.1)	57 (16.0)	1.24 (0.78-1.97)	0.352
C	T	G	40 (12.4)	25 (7.0)	1.96 (1.10-3.47)	0.020

OR: odds ratio; CI: confidence interval; IRSM: idiopathic recurrent spontaneous miscarriage. ^∗^*p* < 0.017 was considered statistically significant after the Bonferroni correction.

## Data Availability

Derived data supporting the findings of this study are available from the corresponding author upon reasonable request.
